# Epidemiology of Malocclusion and Assessment of Orthodontic Treatment Need for Nepalese Children

**DOI:** 10.1155/2014/768357

**Published:** 2014-12-21

**Authors:** Varun Pratap Singh, Amita Sharma

**Affiliations:** ^1^Department of Orthodontics, Nobel Medical College & Teaching Hospital, Biratnagar 56700, Nepal; ^2^Department of Dentistry, SHKM Government Medical College, Mewat, Haryana 122107, India

## Abstract

*Objective.* To evaluate the prevalence of malocclusion and orthodontic treatment needs among 12- to 15-year-old schoolchildren in eastern Nepal and compare the findings with those of other populations. *Methods.* Two thousand seventy-four children (1149 males and 925 females) aged between 12 and 15 years were evaluated. Their orthodontic treatment need was assessed using the Index of Orthodontic Treatment Needs (IOTN) (dental health component (DHC)). Angle's classes of malocclusion were also evaluated. *Results.* The prevalence of classes I, II, and III was 48.50%, 32.68%, and 4.32%, respectively. The IOTN showed that 21.59% had an extreme treatment need, 24.67% had severe treatment need, 24.07% had moderate treatment need, 14.7% had mild treatment need, and 15.02% had no treatment need. *Conclusion.* Class I malocclusion is the most common, while class III is the least prevalent in eastern Nepal. The majority of the children need orthodontic treatment.

## 1. Introduction

Epidemiology of malocclusion and assessment of orthodontic treatment needs are of national importance in many countries and were thus included in numerous national level surveys [1–16]. Malocclusion features the third highest prevalence among oral pathologies, secondarily to dental caries and periodontal disease and therefore ranks third among worldwide public health dental disease priorities. According to World Health Organization the main oral diseases should be subjected to periodic epidemiologic surveys. These assessments are necessary to plan sufficient treatment facilities and develop adequate training programs for respective specialists [17].

Malocclusions can be assessed with various methods [18–23] but not one has gained universal acceptance. The Index of Orthodontic Treatment Need (IOTN) was developed to grade malocclusion on the basis of the significance of various occlusal traits for dental health and esthetic impairment [23]. The IOTN incorporates a dental health component (DHC) based on the recommendations of the Swedish medical board and an esthetic component [24]. Several prevalence studies have been conducted on children in mixed or permanent dentitions [25–33]. However, prevalence of malocclusion and orthodontic treatment needs for Nepalese children had not yet been reported in the indexed literature. Therefore, the present study aims to assess the epidemiology of malocclusion and orthodontic treatment needs in Nepalese schoolchildren aged 12–15 years in Eastern Nepal using IOTN index and compare these data with that of other population.

## 2. Materials and Methods

This study was conducted after ethical clearance from institutional review board, BP Koirala Institute of Health Sciences and permission from concerned school authorities. Consent was obtained from all parents before recording data. The sample comprised two thousand seventy-four children (1149 males and 925 females) from twenty high schools consisting of both public and private schools.

The subjects from the selected schools were included only if their chronological age was 12–15 years and if they were permanent inhabitants of Nepal. Those who were undergoing orthodontic treatment or who had completed orthodontic treatment earlier or suffering from any other systemic diseases were excluded from the study.

The examiner and recording assistant were trained prior to the commencement of the study to ensure reliability. A validation exercise was conducted during the study and subsamples of 10% were reexamined to check intraexaminer variability, which was found to be satisfactory (Kappa value = 0.8). The examination for malocclusion was made according to the molar relationship (Angle) and the criteria laid down by DHC of IOTN. In addition the presence of anterior spacing, a feature overlooked by the DHC was evaluated. The orthodontic treatment need was assessed. All the data was analyzed with SPSS software (Version 16.0 for Windows @ 2007 SPSS INC., NY, USA) and descriptive statistics were calculated.

## 3. Results

Out of 2074 children selected for the study, 14 did not return the signed parental consent document, 45 were not present at school on the assessment day, and 5 had already started orthodontic treatment.

The age distribution of the remaining 2010 children, according to the gender, is presented in Table 1.

Most children exhibited some type of malocclusion. Angle's class I malocclusion was found in 48.50% of all children, class II div. 1 in 29.35%, class II div. 2 in 3.33%, class III in 4.32%, and normal occlusion in 14.42% (Table 2).

Table 3 shows the percentage scores of individual malocclusion traits according to the DHC of the IOTN.

Crowding was the most common type of malocclusion presented by the study group (19.75%) followed by increased overjet (17.51%) and deep overbite (13.23%). Features like scissor bite (0.89%), reverse overjet (1.79%), and open bite (2.03%) were least noticed in the study group.

The IOTN (DHC) showed the following distribution: Grade 1—15.02% (*n* = 302); Grade 2—14.7% (*n* = 296); Grade 3—24.07% (*n* = 484); Grade 4—24.67% (*n* = 496); Grade 5—21.59% (*n* = 432). Grades 3 and 4 were more commonly observed in Nepalese children followed by Grades 5 and 1 (Table 4).

## 4. Discussion

The present study was designed to provide information about the prevalence of malocclusion and orthodontic treatment needs among 12- to 15-year-old school going children. Although assessment of malocclusion in nongrowing population is more reliable, this range was chosen because it represents the majority of schoolchildren with developing malocclusion who require orthodontic treatment. The distribution of malocclusion and treatment needs among schoolchildren in Nepal had not yet been reported in the literature. In our study 14.42% had a normal occlusion, 48.50% had class I, 29.35 had class II div. I, 3.33% had class II div. II, and 4.32% had class III. The frequency of Angle's class I malocclusion in this study was similar to that reported by Proffit et al. [1] in United States but less than Chinese [16] and Turkish [32] populations. The frequency of Angle's class II malocclusion was, however, higher than that being reported in United States [1], Turkish [32], and Chinese populations [16]. In case of class III malocclusion, the frequency was higher than that in United States [1] population but less than that of Chinese [16] and Turkish populations [32]. The difference in the frequency of the various Angle's classes in all these studies can mainly be explained by differences in sample size and ethnicity.

In this study crowding was the most common individual malocclusion trait as in accordance with studies in Maltese [38] and Brazilian [17] populations. The high prevalence of crowding can partially be explained by the great incidence of carious lesions and extractions of deciduous molars which favours migration of the first permanent molars as well as inclinations and rotations. However, scissor bite was the least common malocclusion in this study as in Maltese [38] population while in Brazilian population [17] ankylosed deciduous teeth were the least common trait.

Table 5 compares the finding of dental health component of IOTN in various studies in different populations.

The present investigation showed a greater frequency of Grades 4 and 5 which is severe and extreme treatment needs. This is due to a greater frequency of impacted, submerged deciduous teeth and hypodontia; further differences in sample size, study design, and ethnicities of the sample may account for differences in result.

One can note that the same type of malocclusion falls into different levels of orthodontic treatment need according to its severity. Therefore, the degree and priority of orthodontic treatment need among populations, which are important factors in public health planning, cannot be fully known by just evaluating the malocclusion prevalence [39]. If no specific index is used, determination of who really needs treatment becomes difficult and arbitrary, particularly among dentists and pediatric dentists, who end up inappropriately referring their patients to orthodontic treatment. In the present study, however, the normative evaluation based on the Index of Orthodontic Treatment Need may not be enough because of the often inherent elective nature of this treatment. As a result, other factors such as perceptual, functional, and social needs may interfere with treatment demand and service planning since those factors does not always coincide with the professional evaluation of treatment need. Therefore, further studies investigating the patient's perception and his or her concern regarding orthodontic treatment should be carried out in order to enhance the IOTN efficacy [40, 41].

## 5. Conclusion

Angle's class I malocclusion is the most common while Angle's class III is the least prevalent malocclusion in eastern Nepalese schoolchildren. The pattern of malocclusion in this population sample is in general similar to that published in the international literature. It differs in the fact that this population have a high number of Grade 1 but a lower number of Grade 2 treatment needs of DHC of IOTN. Further this population exhibits a higher number of Grade 5 treatment needs emphasizing an extreme treatment need.

## Figures and Tables

**Table 1:** Age and gender distribution of the study participants.

Sex	*n*	%	Mean age	SD
Males	1121	55.77	13.9	5.7
Females	889	44.22	12.5	5.7

Total	2010	100	13.5	5.8

Age (in years) and gender distribution. (*n* = number of patients and SD = standard deviation).

**Table 2:** Distribution of malocclusion according to Angle's classification.

Malocclusion	Males	Females	Total	Percentage
Angle's class I	557	418	975	48.50
Angle's class II div. I	340	250	590	29.35
Angle's class II div. II	38	30	68	3.33
Angle's class III	31	56	87	4.32
Normal occlusion	155	135	290	14.42

Total	1121	889	2010	100

**Table 3:** Prevalence (%) and distribution of individual malocclusion traits as per DHC of IOTN.

Individual malocclusion traits	Males	Females	Total	%
Increased overjet	192	160	352	17.51
Reverse overjet	21	15	36	1.79
Crossbite	53	39	92	4.57
Deep overbite	141	125	266	13.23
Open bite	21	20	41	2.03
Scissor bite	11	7	18	0.89
Crowding mild	30	23	53	19.75
Moderate	69	61	130	
Severe	132	82	214	
Hypodontia	54	26	80	3.90
Impacted teeth	72	44	116	5.77
Submerged deciduous teeth	41	31	72	3.50
Supernumerary teeth	28	34	62	3.08
Anterior spacing	89	87	176	8.75
Normal occlusion	167	135	302	15.02

Total	1121	889	2010	100

**Table 4:** Relationship between the IOTN (DHC) grades and study population.

IOTN (DHC)	Males	Females	Total	Percentage
Grade 1—no need for treatment	161	141	302	15.02
Grade 2—mild/little need	159	137	296	14.7
Grade 3—moderate/borderline need	292	192	484	24.07
Grade 4—severe treatment need	251	245	496	24.67
Grade 5—extreme treatment need	258	174	432	21.59

Total	1121	889	2010	100

**Table 5:** The data for various IOTN studies as compared to the present study in terms of IOTN (DHC) grades.

Study	Grade 1	Grade 2	Grade 3	Grade 4	Grade 5
Brook and Shaw (United Kingdom), 1989 [34]	7.2%	27.92%	32.13%	27.62%	5.10%
So and Tang (Turkey), 1993 [35]	2%	21%	25%	49%	3%
Burden and Holmes (United Kingdom), 1994 [36]	5.03%	40.96%	24.02%	20.02%	9.45%
Hamdan (Jordan), 2001 [37]	9.3%	40.93%	22.18%	20.31%	7.18%
Camilleri and Mulligan (Malta), 2007 [38]	13.96%	15.09%	28.86%	25.84%	16.22%
Current study (Nepal), 2013	15.02%	14.7%	24.07%	24.67%	21.59%
